# Acentuada Hipertrofia Miocárdica Concêntrica, com Boa Evolução Pós-operatória, em Criança com 4 Anos de Idade

**DOI:** 10.36660/abc.20220869

**Published:** 2023-06-07

**Authors:** Edmar Atik, Gabriela Nunes Leal, Marcelo B. Jatene

**Affiliations:** 1 Hospital Sírio Libanês de São Paulo São Paulo SP Brasil Hospital Sírio Libanês de São Paulo , São Paulo , SP – Brasil

**Keywords:** Anormalidades Congênitas/cirurgia, Cardiomiopatia Hipertrófica/cirurgia, Hipertrofia Ventricular Esquerda/cirurgia, Morte Súbita Cardíaca, Fatores de Risco, Diagnóstico por Imagem/métodos

## Introdução

A miocardiopatia hipertrófica (MCH), caracterizada por hipertrofia do ventrículo esquerdo, não encontra explicação etiológica sob condições das causas usuais que originam sobrecarga cardíaca decorrente da hipertensão arterial sistêmica, cardiopatias congênitas obstrutivas e de outras patologias. ^1 - 5^ Decorre de patologia genética cardiovascular ocasionando doença complexa, desde a diversidade gênica (para a qual já foram identificadas mais de 1400 mutações, em 11 genes diferentes), à expressão fenotípica, às características histológicas e à sintomatologia manifestada. Os genes para beta miosinas *(MYH7)* ocorrem em 35% dos casos e miosina ligada à proteína C (MYBPC3) em 49%, correspondendo a três quartos das mutações patogênicas. ^4^ Maior risco evolutivo é observado no primeiro grupo de alterações, principalmente à morte súbita. O desarranjo de miofibrilas com núcleos bizarros e aumento do tecido conjuntivo extracelular caracterizam os achados histológicos. ^4^

O diagnóstico genético é alcançado em 80% desses casos em crianças, importante para a estratificação de risco, do planejamento terapêutico e o aconselhamento genético. ^3^

Os sintomas da cardiomiopatia hipertrófica podem incluir a respiração encurtada, especialmente durante a prática de exercícios, dores no peito, desmaios, fadiga, sensação de batimento cardíaco acelerado ou palpitações, sopro cardíaco decorrente de obstruções ao fluxo e da insuficiência mitral correlata. ^2 - 4^

A morte súbita cardíaca é a consequência mais imprevisível e temida da MCH, e ocorre predominantemente em jovens, em indivíduos assintomáticos ou mesmo com sintomatologia frustra. ^4 - 7^ A prevenção de riscos súbitos deve ser orientada a intervenção cirúrgica, após tratamento clínico ineficaz e após a devida avaliação por ecocardiograma com esforço físico. ^8^ É reconhecida também a eficácia exclusiva do cardioversor desfibrilador implantável (CDI) na prevenção da morte súbita. ^9^ Na abordagem aos pacientes com MCH e respectivas famílias, torna-se assim fundamental a correta avaliação do risco de morte súbita e do potencial benefício da implantação desse dispositivo em prevenção primária. ^7 - 9^

A insuficiência cardíaca diastólica ocorre em 36% dos pacientes, que se beneficiam com o uso de betabloqueadores adrenérgicos. Essa medicação, dada em alta dose (> 4,5 mg/Kg/dia) torna-se primordial no aumento da sobrevida desses pacientes, que passa de 54% para 93% após 5 anos de evolução e propicia melhora da função diastólica, redução da obstrução da via de saída ventricular, aumento da cavidade ventricular e aumento do débito cardíaco. ^6 , 10^

Betabloqueadores são assim as principais drogas no tratamento farmacológico da cardiomiopatia hipertrófica. Aliviam os sintomas em 2/3 dos pacientes e reduzem a obstrução da via de saída do ventrículo esquerdo durante o esforço físico, sendo, portanto, a droga de escolha nesses pacientes. Demonstra-se também a efetividade da disopiramida, inotrópico negativo por bloqueio dos canais de sódio, no alívio da obstrução do trato de saída do ventrículo esquerdo, embora pareça que diminua seu efeito com o tempo. ^11^

Importa recordar as recomendações da *American College of Cardiology Foundation e American Heart Association* nesta patologia desde 2011 em relação ao diagnóstico, tratamento e aspectos preventivos, principalmente da morte súbita. ^5 - 7^ São representados por pelo menos um desses elementos **:** espessura máxima da parede do ventrículo esquerdo ≥ 30 mm, síncope inexplicada, taquicardia ventricular não sustentada, história familiar de morte súbita e resposta anormal da pressão arterial durante o exercício. Outros fatores de risco clássicos combinados também são considerados como o gradiente de pressão na via de saída do ventrículo esquerdo, o diâmetro do átrio esquerdo e a idade. ^5 - 7^

Dentre os pacientes com MCH, destaca-se que um terço deles manifestam obstrução da via de saída de ventrículo esquerdo, em um terço essa obstrução se manifesta ao exercício e no outro terço evoluem sem qualquer obstrução. ^10^

Gravidade maior ocorre em exteriorização precoce no primeiro ano de vida e o risco a partir daí se assemelha ao do adulto, em cerca de 1% de malefícios ao ano. ^9^

O objetivo deste trabalho consiste em demonstrar o tratamento cirúrgico de miectomia septal como procedimento de realce na melhora dos sintomas e na prevenção de morte súbita, na comparação com o mesmo procedimento na literatura médica.

## Descrição do caso

### Dados clínicos

Criança do sexo feminino com 4 anos de idade, hígida até desenvolver síncope após ter subido rapidamente dois lances de escada há um mês, quadro que se repetiu no dia seguinte. Retrospectivamente, certo cansaço discreto era notado pela mãe comparando-a com sua irmã normal, mesmo gêmea univitelina. Sopro cardíaco discreto havia sido auscultado há dois anos em vigência de infecção viral. Na ocasião, o ecocardiograma havia salientado discreta alteração hipertrófica no septo ventricular. Ecocardiograma atual revelou a hipertrofia miocárdica concêntrica com predomínio septal e obstrução acentuada da via de saída do ventrículo esquerdo e com insuficiência valvar mitral. Medicação betabloqueadora foi iniciada a seguir, com propranolol- 40 mg/dia.

Exame físico: bom estado geral, eupneica, acianótica, pulsos normais nos quatro membros. Peso: 19 Kg, Alt.: 100 cm, PA: 80/50 mm Hg, FC: 90 bpm, saturação de oxigênio=98%. Aorta não palpada na fúrcula.

Precórdio: *ictus cordis* não palpado, sem impulsões sistólicas na borda esternal esquerda. Bulhas cardíacas normofonéticas, sopro sistólico rude de moderada intensidade ++/4, de ejeção, mais audível na borda esternal esquerda e na área mitral. Fígado não palpado e pulmões limpos.

### Exames Complementares

**Figura 1:**
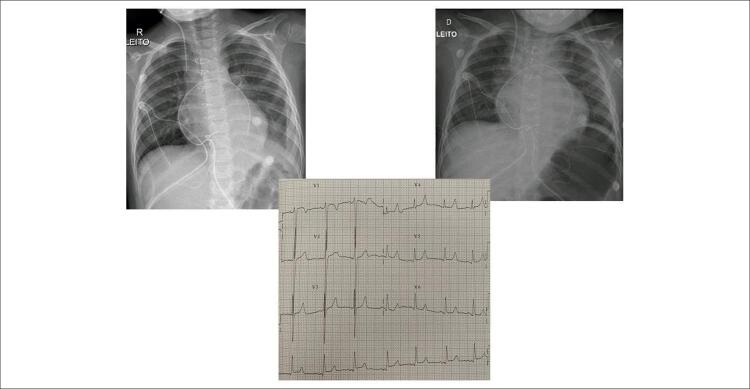
– Radiografias de tórax em período pós-operatório imediato salientam a forma globosa do coração, decorrente da hipertrofia miocárdica e o ECG no plano horizontal mostra sinais da sobrecarga ventricular esquerda com repolarização ventricular normal.

**Eletrocardiograma:** ritmo sinusal, PR: 0,12, QRS: 0,93, QTc: 0,43, sinais de sobrecarga ventricular esquerda com ondas S profundas em V1-V3 e índice de Sokolof de 38 mm. Morfologia rS em V1 e qR em V6. Não havia alterações da repolarização ventricular. AP= +60 ^o^ , AQRS= +45 ^o^ , AT= +35 ^o^ . Demonstração no plano horizontal ( Figura 1 ).

**Radiografia de tórax:** área cardíaca com limite normal (índice cardiotorácico=0,50) com morfologia arredondada e globosa, arco aórtico normal e trama vascular pulmonar normal, em duas imagens em período pós-operatório imediato ( Figura 1 ).

**Figura 2:**
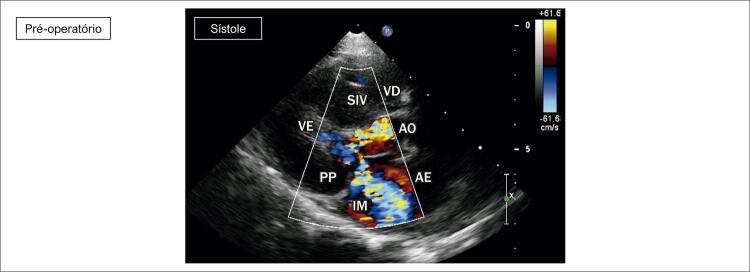
– Corte paraesternal eixo longo, evidenciando obstrução ao fluxo em via de saída de ventrículo esquerdo e insuficiência mitral. SIV: septo interventricular; PP: parede posterior; VE: ventrículo esquerdo; AO: aorta; AE: átrio esquerdo; VD: ventrículo direito; IM: insuficiência mitral.

**Figura 3 f03:**
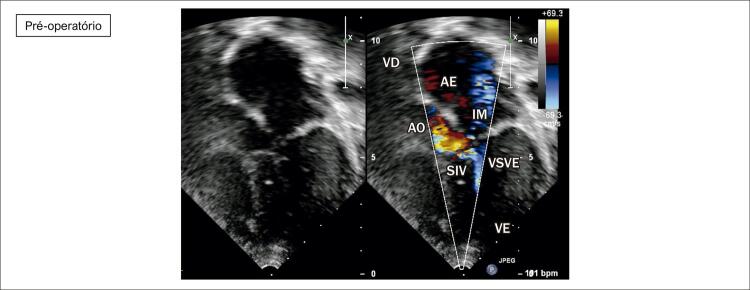
– Corte apical cinco câmaras, evidenciando obstrução ao fluxo em via de saída de ventrículo esquerdo e insuficiência mitral. SIV: septo interventricular; VD: ventrículo direito; VE: ventrículo esquerdo; AO: aorta; AE: átrio esquerdo; VSVE: via de saída de ventrículo esquerdo; IM: insuficiência mitral.

**Figura 4 f04:**
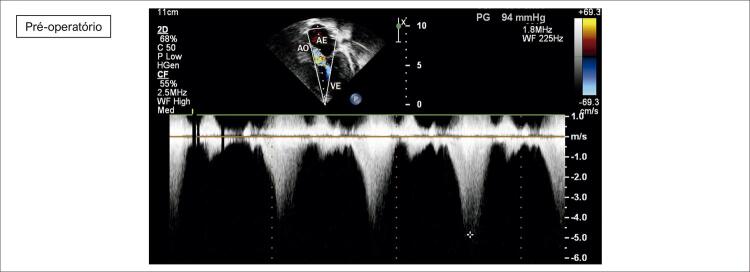
– Corte apical cinco câmaras, evidenciando obstrução ao fluxo em via de saída de ventrículo ao color Doppler. Gradiente máximo obtido em via de saída ventricular = 94 mmHg. AO: aorta; AE: átrio esquerdo; VE: ventrículo esquerdo.

**Figura 5 f05:**
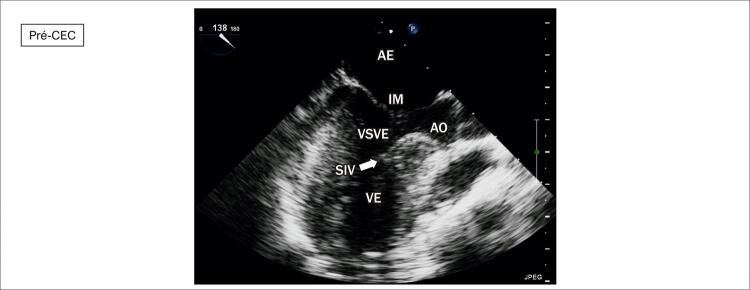
– Ecocardiograma intraoperatório pré circulação extracorpórea. VE: ventrículo esquerdo; SIV: septo interventricular; AO: aorta; VSVE: via de saída de ventrículo esquerdo; IM: insuficiência mitral; AE: átrio esquerdo.

**Figura 6 f06:**
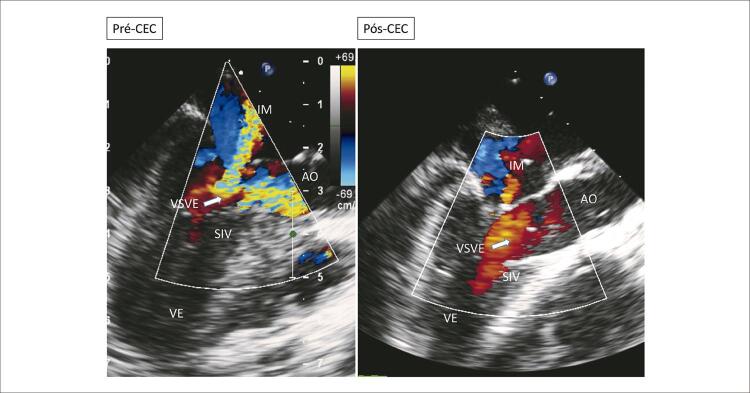
– Ecocardiograma transesofágico intraoperatório pré e pós saída de circulação extracorpórea (CEC). VE: ventrículo esquerdo; SIV: septo interventricular; AO: aorta; VSVE: via de saída de ventrículo esquerdo; IM: insuficiência mitral.

**Figura 7 f07:**
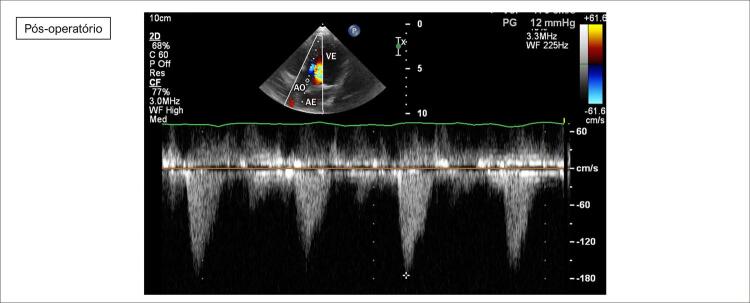
– Corte apical cinco câmaras, demonstrando redução do gradiente em via de saída de ventrículo esquerdo ao color Doppler (12 mmHg) após miectomia. VE: ventrículo esquerdo; AE: átrio esquerdo; AO: aorta.

**Ecocardiograma** : As cavidades cardíacas eram normais, sendo VE=27, AE=29, VD=18, Ao=15, FEVE=88%, septo ventricular=13, parede posterior de VE= 9 mm. Obstrução hipertrófica do septo ventricular basal com gradiente máximo de 87 mm Hg ( Figuras 2-7 [Fig f03] [Fig f04] [Fig f05] [Fig f06] [Fig f07] ).

**Diagnóstico clínico:** miocardiopatia hipertrófica concêntrica com predomínio do septo ventricular com obstrução acentuada da via de saída de ventrículo esquerdo e insuficiência mitral acentuada. Episódios recentes de síncope ao esforço físico.

### Características clínicas

1. ***Raciocínio Clínico:*** nesta criança sem sintomas prévios e com síncope recente ao esforço físico, os elementos clínicos orientaram para o diagnóstico presumível de miocardiopatia hipertrófica com obstrução da via de saída de ventrículo esquerdo, denotado pelo sopro sistólico rude e intenso ao longo da borda esternal esquerda. Acresce a forma globosa do coração na radiografia de tórax. Essa impressão foi consolidada pelo ecocardiograma, em uma demonstração nítida da hipertrofia miocárdica concêntrica com predomínio do septo ventricular, que obstruía a via de saída do ventrículo esquerdo.
2. ***Diagnóstico diferencial:*** em paciente com síncope, diagnóstico de possível arritmia cardíaca decorrente de alguma canulopatia, deve também ser lembrada, assim como a possibilidade de compressão coronária por anomalia de origem e trajeto coronário anômalo. Tais possibilidades, no entanto, foram afastadas em face da presença do sopro sistólico que denunciou a obstrução ao fluxo sanguíneo.

**Conduta:** Dada a repercussão da MCH com obstrução, insuficiência mitral e síncope prévia, a orientação imediata foi o alívio cirúrgico dos defeitos. Por esternotomia mediana o timo foi preservado, circulação extracorpórea estabelecida pela aorta e duas veias cavas, em temperatura de 30 ^o^ C. O átrio direito foi aberto assim como o septo atrial para descompressão das cavidades esquerdas. Pela aortomia, a valva aórtica era normal e se visualizou a presença de acentuada hipertrofia septal na via de saída do ventrículo esquerdo. Através a miectomia dessa região houve alívio acentuado da obstrução. Tempo de CEC de 65 minutos e de anóxia de 42 minutos. Na saída de CEC, observadas extrassístoles supraventriculares frequentes.

Ecocardiograma transoperatório revelou a MCH concêntrica acentuada com predomínio septal. O segmento basal do septo era de 15 mm de espessura com gradiente máximo na via de saída do ventrículo esquerdo de 87 mm Hg. Havia movimento sistólico anterior da valva mitral e insuficiência mitral acentuada. Após a miectomia septal da região basal, o gradiente máximo se tornou de 4 mm Hg e a insuficiência mitral discreta com dois jatos pequenos. Houve preservação da função ventricular com a continuidade da alteração do relaxamento ventricular esquerdo.

Na evolução imediata houve desaparecimento do sopro sistólico da borda esternal esquerda. A frequência cardíaca mais baixa no pós-operatório imediato de cerca de 60 bpm aumentou para 80 bpm em ritmo sinusal em uso de Noradrenalina e vasodilatador sistêmico, Milrinona em doses baixas, por três dias. Permaneceu com drenagem exagerada pelos drenos pleural e do mediastino por 4 dias. A radiografia de tórax mostrou o mesmo aspecto do pré-operatório com a forma globosa e o eletrocardiograma também permaneceu inalterado, exceção ao ritmo juncional intercalado com o sinusal. Na restituição da medicação com Propranolol, teve alta em boas condições clínicas, na esperança do controle do quadro sincopal, mas com a continuidade do cuidado evolutivo da hipertrofia miocárdica.

## Discussão

A cardiomiopatia hipertrófica é a segunda forma mais comum de doença do músculo cardíaco que afeta crianças e adolescentes e é a principal causa de morte súbita em jovens atletas. ^12 , 13^ A maioria na infância é causada por mutações nos genes da proteína do sacômero cardíaco. ^1 - 5^ O diagnóstico de MCH em bebês geralmente é feito durante a avaliação de um sopro cardíaco ou em insuficiência cardíaca congestiva. As crianças geralmente são encaminhadas para avaliação de sintomas, anormalidades eletrocardiográficas ou pelo encontro de sopro cardíaco, ou ainda para triagem familiar após diagnóstico de MCH em um parente. Como a maioria dos casos de MCH são familiares, a avaliação de parentes de primeiro grau e outros membros da família em risco de herdar a doença deve ser um componente de rotina do manejo clínico. ^1 - 5^ A estratificação de risco na população pediátrica permanece um desafio.

Recentemente, os avanços no diagnóstico e nas opções de tratamento foram fundamentais para diminuir a frequência de eventos clínicos adversos. Entretanto, a eliminação completa da morte cardíaca súbita ainda permanece o maior ganho e indescritível. Pacientes pediátricos sintomáticos tem alto índice de mortalidade (6%/ano) e a evolução após a miectomia tem sido boa, ^7 - 10^ como também demonstrado no presente caso. Mas, se deve lembrar que o tratamento médico tem sido a conduta inicial para pacientes sintomáticos, mesmo com obstrução do trato de saída do ventrículo esquerdo. ^6 - 9^

Em pacientes sintomáticos sem sinais de obstrução importante da via de saída ventricular se recomenda firmemente a execução de ecocardiograma sob estresse físico, assim como a manutenção em posição ortostática após o exercício como um fator importante na indução da obstrução anteriormente não detectada. ^8^

A miectomia septal para pacientes sintomáticos devido à obstrução detectada da via de saída ventricular continua sendo um tratamento excelente, caso não haja a devida melhora clínica. ^7^

De 1975 a 2003 na *Mayo Clinic, Rochester-Minnesota, USA* , 56 pacientes foram submetidos à miectomia, de 2 meses a 20 anos de idade, em média de 11+5,6 anos. O gradiente de pressão ventricular diminuiu de 103+34 para 16+12 mmHg e em evolução pós-operatória média de 8,6+6,2 anos, e a sobrevida foi de 97 e 93% em 5 e 10 anos respectivamente, estando 96% em CF-I-II. Reoperação ocorreu em 8 pacientes sendo em 2 por nova miectomia septal. ^14^

Experiências recentes na MCH são limitadas e saliento também a boa evolução de pacientes operados com obstrução da via de saída dos dois ventrículos. Em estudo da *Zhejiang University-Hangzhou-China* , desde 2009 a 2018 em 117 crianças consecutivas submetidas a miectomia, de 6 meses a 17 anos, observou-se sobrevida de 100% em 1 ano e 96,5% em 3 anos, excluindo-se três mortes súbitas pós-operatórias. Neste estudo, havia 22 pacientes (18.8%) com obstrução à direita e 61 (52,1%) à esquerda e pontes miocárdicas em 25 (21,4%) pacientes. Houve no pós-operatório evolutivo diminuição do gradiente de pressão obstrutivo assim como da insuficiência mitral e da espessura do septo ventricular. ^15^ Outro estudo em 11 pacientes, operados de 1993 a 2013, na *Mayo Clinic-USA* , com média de idade de 13 anos (2 meses a 28 anos), todos sintomáticos com gradientes de pressão de 60+18 e 78+24 mmHg à direita e à esquerda, obtiveram boa resolução das obstruções e sem riscos evolutivos em tempo médio de 4,6 anos, máximo de 16,3 anos. ^16^

Outra técnica também aplicada é a de *Konno* modificada, especialmente quando há obstrução biventricular e em crianças abaixo de 5 anos. Serviço francês do *Hôpital Universitaire Necker em Paris* mostrou bons resultados em 79 pacientes, operados entre 1991 e 2016, com sobrevida de 82% em 20 anos. ^17^

Como conclusão, o tratamento cirúrgico de lesões obstrutivas causadas pela miocardiopatia hipertrófica, se torna favorável em pacientes selecionados, em vista da real necessidade da intervenção.

Esse procedimento sem dúvida diminui a incidência de morte súbita, de insuficiência cardíaca e da progressão da miocardiopatia hipertrófica. Controle médico constante e rigoroso se impõe assim como instituição de hábitos de atividade física restrita e ainda sem a necessidade de profilaxia antibiótica.
