# Supplementary material for: Acentuada Hipertrofia Miocárdica Concêntrica, com Boa Evolução Pós-operatória, em Criança com 4 Anos de Idade
Source: Arq Bras Cardiol. 2023 Jun 7;120(5):e20220869. [Article in Portuguese] doi: 10.36660/abc.20220869 (PMC12092034; doi:10.36660/abc.20220869)
Supplement: Translation

# Translation: Marked Concentric Myocardial Hypertrophy with Good Postoperative Evolution in a 4 Years Old Child

**Authors:** Edmar Atik, Gabriela Nunes Leal, Marcelo B. Jatene

**Affiliations:** 1 Brazil Hospital Sírio Libanês de São Paulo, São Paulo, SP – Brazil

**Keywords:** Abnormalities Congenital/surgery, Cardiomyopathy, Hypertrophic/surgery, Hypertrophy, Left Ventricular/surgery, Sudden Cardiac Death, Risk Factors, Diagnostic Imaging/methods

## Introduction

Hypertrophic cardiomyopathy (HCM), characterized by hypertrophy of the left ventricle, has no etiological explanation under conditions of the usual diseases that cause cardiac overloads like systemic arterial hypertension, obstructive congenital heart diseases, and other pathologies.^1 - 5^It results from cardiovascular genetic pathology causing a complex disease, from gene diversity (more than 1400 mutations identified in 11 different genes) to phenotypic expression, histological characteristics, and manifested symptoms. Beta myosins (MYH7) genes occur in 35% of the cases, and myosin is linked to protein C (MYBPC3) in 49%, corresponding to three-quarters of the pathogenic mutations.^4^Higher evolutionary risk is observed in the first group of changes, mainly to sudden death. The breakdown of myofibrils with bizarre nuclei and increased extracellular connective tissue characterize the histological findings.^4^

Genetic diagnosis is achieved in 80% of these cases in children, which is important for risk stratification, therapeutic planning, and genetic counseling.^3^

Symptoms of hypertrophic cardiomyopathy may include shortness of breath, especially during exercise, chest pain, fainting, fatigue, a feeling of rapid heartbeat or palpitations, heart murmur due to flow obstructions, and related mitral regurgitation.^2 - 4^

Sudden cardiac death is the most unpredictable and feared consequence of HCM and occurs predominantly in young people, asymptomatic individuals, or even with frustrated symptoms.^4 - 7^Preventing sudden risks should be oriented towards surgical intervention after ineffective clinical treatment and due assessment by echocardiogram with physical effort.^8^The unique effectiveness of the implantable defibrillator cardioverter (ICD) in preventing sudden death is also recognized.^9^In the approach to patients with HCM and their families, it is essential to correctly assess the risk of sudden death and the potential benefit of implanting this device in primary prevention.^7 - 9^

Diastolic heart failure occurs in 36% of patients who benefit from adrenergic beta-blockers. This medication, given in a high dose (> 4.5 mg/kg/day), is essential in increasing the survival of these patients, which goes from 54 to 93% after 5 years of evolution. It improves diastolic function, reduces ventricular outflow tract obstruction, and increases the ventricular cavity and cardiac output.^6 , 10^

Beta-blockers are thus the main drugs in the pharmacological treatment of hypertrophic cardiomyopathy. They relieve symptoms in 2/3 of patients and reduce left ventricular outflow tract obstruction during physical exertion, thus being the drug of choice in these patients. It also demonstrates the effectiveness of disopyramide, a negative inotrope by blocking sodium channels, in relieving left ventricular outflow tract obstruction, although it seems to decrease its effect over time.^11^

It is important to remember the recommendations of the American College of Cardiology Foundation and American Heart Association in this pathology since 2011 concerning diagnosis, treatment, and preventive aspects, mainly of sudden death.^5 - 7^They are represented by at least one of these elements: maximum left ventricular wall thickness ≥ 30 mm, unexplained syncope, non-sustained ventricular tachycardia, family history of sudden death, and abnormal blood pressure response during exercise. Other classic risk factors combined are also considered, such as the pressure gradient in the left ventricular outflow tract, the diameter of the left atrium, and age.^5 - 7^

Among HCM patients, it is highlighted that one-third of them manifest obstruction of the left ventricular outflow tract; in one-third, this obstruction is manifested during exercise, and in the other third, they evolve without any obstruction.^10^

Greater severity occurs in early externalization in the first year of life, and the risk after that is similar to that of adults, with about 1% of harm per year.^9^

This work aims to demonstrate the surgical treatment of septal myectomy as an enhancement procedure in improving symptoms and preventing sudden death compared to the same procedure in the medical literature.

## Case description

### Clinical data

Female child, 4 years old, healthy until she developed syncope after having quickly climbed two flights of stairs a month ago, a picture repeated the next day. In retrospect, the mother noticed slight discreet tiredness, comparing her to the normal sister, even a twin sister. A mild heart murmur had been heard two years ago in the presence of a viral infection. At the time, the echocardiogram had highlighted a slight hypertrophic change in the ventricular septum. The current echocardiogram revealed concentric myocardial hypertrophy with septal predominance, marked left ventricular outflow tract obstruction, and mitral valve insufficiency. Beta-blocking medication was started next, with propranolol- 40 mg/day.

Physical examination: good general condition, eupneic, acyanotic, normal pulses in the four limbs. Weight: 19 Kg, Height: 100 cm, BP: 80/50 mm Hg, HR: 90 bpm, oxygen saturation = 98%. Aorta is not palpated at the suprasternal notch.

Precordium: apical impulse not palpable, without systolic impulses at the left sternal border. Normal intensity heart sounds, a rough systolic murmur of moderate intensity ++/4, ejection, more audible on the left sternal border and in the mitral area. Liver not palpated, and lungs clear.

### Complementary exams

**Electrocardiogram:** sinus rhythm, PR: 0.12, QRS: 0.93, QTc: 0.43, signs of left ventricular overload with deep S waves in V1-V3 and 38 mm Sokolof index. RS morphology in V1 and qR in V6. There were no changes in ventricular repolarization. AP = +60^o^, AQRS= +45^o^, AT = +35^o^. Demonstration in the horizontal plane ( Figure 1 ).

**Figure 1:** – Chest radiographs in the immediate postoperative period highlight the globose shape of the heart, resulting from myocardial hypertrophy, and the ECG in the horizontal plane shows signs of left ventricular overload with normal ventricular repolarization.

**Chest radiography:** cardiac area with normal limit (cardiothoracic index = 0.50) with rounded and globular morphology, normal aortic arch, and normal pulmonary vascular network in two images in the immediate postoperative period ( Figure 1 ).

**Echocardiogram:** Cardiac cavities were normal, LV = 27, LA = 29, RV = 18, Ao = 15, LVEF = 88%, ventricular septum=13, LV posterior wall=9 mm. Hypertrophic obstruction of the basal ventricular septum with a maximum gradient of 87 mm Hg ( Figures 2-7 [Fig f03001] [Fig f04001] [Fig f05001] [Fig f06001] [Fig f07001] ).

**Figure 2:** – Parasternal long-axis view, showing obstruction to flow in the left ventricular outflow tract and mitral regurgitation. VS: interventricular septum; PP: posterior wall; LV: left ventricle; AO: aorta; LA: left atrium; RV: right ventricle; MI: mitral insufficiency.

**Figure 3 f03001:** – Apical five-chamber view, showing obstruction to flow in the left ventricular outflow tract and mitral insufficiency. VS: interventricular septum; RV: right ventricle; LV: left ventricle; AO: aorta; LA: left atrium; LVO: left ventricular outflow tract; MI: mitral insufficiency.

**Figure 4 f04001:** – Apical five-chamber view, showing obstruction to flow in the outflow tract of the ventricle on color Doppler. Maximum gradient obtained in the ventricular outflow tract = 94 mmHg. Ao: aorta; LA: left atrium; LV: left ventricle.

**Figure 5 f05001:** – Intraoperative echocardiogram before cardiopulmonary bypass. LV: left ventricle; VS: interventricular septum; AO: aorta; LVO: left ventricular outflow tract; MI: mitral insufficiency; LA: left atrium.

**Figure 6 f06001:** – Intraoperative transesophageal echocardiogram before and after off-pump cardiopulmonary bypass (CPB). LV: left ventricle; VS: interventricular septum; AO: aorta; LVO: left ventricular outflow tract; MI: mitral insufficiency.

**Figure 7 f07001:** – Apical five-chamber view, demonstrating a reduction in the left ventricular outflow tract gradient on color Doppler (12 mmHg) after myectomy. LV: left ventricle; LA: left atrium; AO: aorta.

**Clinical Diagnosis:** concentric hypertrophic cardiomyopathy with a predominance of the ventricular septum, marked left ventricular outflow tract obstruction, and mitral insufficiency—recent episodes of syncope with physical exertion.

### Clinical characteristics

- **Clinical Reasoning:** in this child without previous symptoms and with recent syncope to physical exertion, the clinical elements guided the presumptive diagnosis of hypertrophic cardiomyopathy with obstruction of the left ventricular outflow tract, denoted by the severe systolic murmur along the left sternal border. Plus, the globose heart shape on the chest X-ray. The echocardiogram consolidated this impression, which clearly demonstrated concentric myocardial hypertrophy with a predominance of the ventricular septum, which obstructed the left ventricular outflow tract.
- **Differential diagnosis:** in a patient with syncope, a diagnosis of possible cardiac arrhythmia due to some canulopathy should also be remembered, as well as the possibility of coronary compression due to an anomaly of origin and also an anomalous coronary path. Such possibilities, however, were ruled out due to the presence of the systolic murmur denouncing an obstruction to blood flow.

**Management:** Given the repercussion of HCM with obstruction, mitral regurgitation, and previous syncope, immediate guidance was the surgical relief of the defect. The thymus was preserved by median sternotomy, and a cardiopulmonary bypass was established through the aorta and two vena cava at a temperature of 30^o^C. The right atrium and the atrial septum were opened to decompress the left cavities. Through the aortomy, the aortic valve was normal and marked septal hypertrophy was seen in the left ventricular outflow tract. Through the myectomy of this region, there was marked relief of the obstruction. CPB time of 65 minutes and anoxia of 42 minutes. At the exit of CPB, frequent supraventricular extrasystoles were observed.

A transoperative echocardiogram revealed marked concentric HCM with septal predominance. The basal segment of the septum was 15 mm thick with a maximum gradient in the left ventricular outflow tract of 87 mm Hg. There was an anterior systolic movement of the mitral valve and marked mitral regurgitation. After septal myectomy of the basal region, the maximum gradient became 4 mm Hg and mild mitral regurgitation with two small jets. The ventricular function was preserved with the continuation of the change in left ventricular relaxation.

In the immediate evolution, the systolic murmur of the left sternal border disappeared. In the immediate postoperative period, the lower heart rate of about 60 bpm increased to 80 bpm in sinus rhythm using Noradrenaline and systemic vasodilator, Milrinone, in low doses, for three days. It remained with exaggerated drainage through the pleural and mediastinal drains for 4 days. The chest X-ray showed the same aspect of the preoperative with the globose shape, and the electrocardiogram also remained unchanged, except for the junctional rhythm interspersed with the sinus. When returning the medication with Propranolol, she was discharged in good clinical condition, hoping to control the syncopal condition but with the continuity of the evolutionary care of myocardial hypertrophy.

## Discussion

Hypertrophic cardiomyopathy is the second most common form of heart muscle disease that affects children and adolescents and is the main cause of sudden death in young athletes.^12 , 13^Mutations in the genes of the cardiac sarcomere protein1-5 cause the majority in childhood. The diagnosis of HCM in infants is usually made while assessing a heart murmur or congestive heart failure. Children are usually referred for symptom assessment, electrocardiographic abnormalities, a heart murmur, or family screening after diagnosis of HCM in a relative. As the majority of cases of HCM are familial, evaluating first-degree relatives and other family members at risk of inheriting the disease should be a routine component of clinical management.^1 - 5^Risk stratification in the pediatric population remains a challenge.

Recently, advances in diagnosis and treatment options have been instrumental in decreasing the frequency of adverse clinical events. However, completely eliminating sudden cardiac death remains the greatest and indescribable gain. Symptomatic pediatric patients have a high mortality rate (6%/year), and the evolution after myectomy has been good,^7 - 10^as demonstrated in the present case. However, remember that medical treatment has been the initial approach for symptomatic patients, even with left ventricular outflow tract obstruction.^6 - 9^

In symptomatic patients without signs of major ventricular outflow tract obstruction, performing an echocardiogram under physical stress is strongly recommended. Maintaining an orthostatic position after exercise is an important factor in the induction of previously undetected obstruction.^8^

The septal myectomy for symptomatic patients due to the detected ventricular outflow tract obstruction remains an excellent treatment if there is no clinical improvement.^7^

From 1975 to 2003, at the Mayo Clinic, Rochester-Minnesota, USA, 56 patients underwent myectomy, from 2 months to 20 years of age, with an average of 11 + 5.6 years. The ventricular pressure gradient decreased from 103 + 34 to 16 + 12 mmHg, the mean postoperative evolution was 8.6 + 6.2 years, and survival was 97 and 93% in 5 and 10 years, respectively, 96% in CF-I-II. Reoperation occurred in 8 patients, 2 due to a new septal myectomy.^14^

Recent experiences in HCM are limited, and I also highlight the good evolution of patients operated on with obstruction of the outlet of both ventricles. In a study by Zhejiang University-Hangzhou-China, from 2009 to 2018, in 117 consecutive children going myectomy from 6 months to 17 years, there was a 100% survival in 1 year and 96.5% in 3 years, excluding three sudden postoperative deaths. In this study, there were 22 patients (18.8%) with obstruction on the right and 61 (52.1%) on the left and myocardial bridges in 25 (21.4%) patients. In the postoperative period, there was a decrease in the obstructive pressure gradient, mitral regurgitation, and ventricular septum thickness.^15^Another study on 11 patients, operated from 1993 to 2013, at Mayo Clinic-USA, with a mean age of 13 years (2 months to 28 years), all symptomatic with pressure gradients of 60 + 18 and 78 + 24 mmHg at right and left, they obtained good resolution of the obstructions and without evolutionary risks in an average time of 4.6 years, maximum of 16.3 years.^16^

Another technique also applied is modified Konno, especially when there is biventricular obstruction and in children under 5 years of age. French service of the Hôpital Universitaire Necker in Paris showed good results in 79 patients operated on between 1991 and 2016, with 82% survival in 20 years.^17^

In conclusion, the surgical treatment of obstructive lesions caused by hypertrophic cardiomyopathy is favorable in selected patients because of the real need for the intervention.

This procedure undoubtedly reduces the incidence of sudden death, heart failure, and the progression of hypertrophic cardiomyopathy. Constant and rigorous medical control is necessary, as well as the establishment of restricted physical activity habits without the need for antibiotic prophylaxis.
